# *Rickettsia rickettsii* in *Amblyomma patinoi* Ticks, Colombia

**DOI:** 10.3201/eid2013.140721

**Published:** 2015-03

**Authors:** Álvaro A. Faccini-Martínez, Francisco B. Costa, Tatiana E. Hayama-Ueno, Alejandro Ramírez-Hernández, Jesús A. Cortés-Vecino, Marcelo B. Labruna, Marylin Hidalgo

**Affiliations:** Pontificia Universidad Javeriana, Bogotá, Colombia (Á.A. Faccini-Martínéz, M. Hidalgo);; Universidade de São Paulo, São Paulo, Brazil (F.B. Costa, T.E. Hayama-Ueno, M.B. Labruna);; Universidad Nacional de Colombia, Bogotá (A. Ramírez-Hernández, J.A. Cortés-Vecino)

**Keywords:** Rickettsia rickettsii, Amblyomma patinoi, Amblyomma cajennense sensu lato, Rocky Mountain spotted fever, isolation, Colombia, ticks, rickettsia, vector-borne infections, bacteria

**To the Editor:** *Rickettsia rickettsii* is the etiologic agent of Rocky Mountain spotted fever (RMSF), a highly lethal tick-borne rickettsioses restricted to the Western Hemisphere (*1*,*2*). In Colombia, *R. rickettsii* was first reported during the 1930s, when 62 (95%) of 65 affected persons died of RMSF in Tobia town (Cundinamarca Department) (*3*), from where highly virulent strains of *R. rickettsii* were isolated through the inoculation of patient blood or of *Amblyomma* *cajennense* sensu lato (s.l.) extracts into guinea pigs (*4*). Thereafter, RMSF remained unnoticed in Colombia until the 21st century, when new outbreaks with high case-fatality rates were reported in different regions, including Villeta, a nearby locality of Tobia (*1*).

Recent studies have shown that *A. cajennense* s.l., widely distributed from the southern United States to Argentina, is actually a complex of 6 different species: *A. cajennense* sensu stricto (Amazonian region), *A. mixtum* (from Texas, USA, to western Ecuador), *A. sculptum* (northern Argentina, Bolivia, Paraguay, Brazil), *A. interandinum* (inter-Andean valley of Peru), *A. tonelliae* (dry areas of northern Argentina, Bolivia, and Paraguay), and *A. patinoi* (eastern cordillera of Colombia) (*5*). With this new classification, *A. patinoi*, originally described from Villeta, is the only species of this complex known to occur in the RMSF-endemic area of Cundinamarca, Colombia (*5*).

In August 2013, we collected 15 *A. patinoi* adult ticks from cattle in Naranjal village (5°3′31.52′′N, 74°26′50.24′′W), Villeta town, an area of Cundinamarca, Colombia, to which RMSF is endemic. Ticks were taken alive to the laboratory, where they were frozen at –80°C for further analysis. The 15 ticks were defrosted, surface sterilized with iodine alcohol, and processed individually by the shell vial technique for isolation of rickettsiae in Vero cells, as described (*6*). Infected cells were always incubated at 28°C. Rickettsiae were observed by Gimenez staining within cells (Technical Appendix Figure 1) from only 1 (inoculated from a female tick) of the 15 inoculated shell vials. This isolate was subjected to at least 7 Vero cell passages, each achieving >90% infected cells.

DNA was extracted from an aliquot of first passage–infected cells and tested by a battery of PCR protocols targeting fragments of the rickettsial genes *gltA*, *ompA*, and *ompB* and the intergenic regions RR0155-*rpmB*, RR1240-*tlc5^b^*, and *cspA-ksgA* (Table). We sequenced 1,106 bp, 512 bp, and 799 bp of the *gltA*, *ompA*, and *ompB* genes, respectively. By BLAST analyses (http://www.ncbi.nlm.nih.gov/blast), these sequences were 100% identical to corresponding sequences of *R. rickettsii* from Colombia and Brazil (GenBank accession nos. CP003306, CP003305). Generated sequences for 2 intergenic regions, RR0155-*rpmB* (228 bp) and RR1240-*tlc5^b^* (306 bp), were 100% identical to corresponding sequences of the same 2 *R. rickettsii* isolates from Colombia and Brazil. A 337-bp sequence of the *cspA-ksgA* intergenic region was 100% (337/337 nt) identical to *R. rickettsii* from Brazil (CP003305) and 99.7% (336/337) to *R. rickettsii* from Colombia (CP003306). Partial sequences from *R. rickettsii* generated in this study were deposited into GenBank and assigned nucleotide accession nos. KJ735644–KJ735649.

**Table:** Primer pairs used for amplification of rickettsial genes or intergenic regions, Colombia, August 2013

Target, primer pairs, primers	Primer sequences, 5′ → 3′	Fragment size, bp	Reference
*gltA*			
1			
CS-78	GCAAGTATCGGTGAGGATGTAAT	401	(*6*)
CS-323	GCTTCCTTAAAATTCAATAAATCAGGAT		(*6*)
2			
CS-239	GCTCTTCTCATCCTATGGCTATTAT	834	(*6*)
CS-1069	CAGGGTCTTCGTGCATTTCTT		(*6*)
*ompA*			
3			
Rr190.70p	ATGGCGAATATTTCTCCAAAA	530	(*7*)
Rr190.602n	AGTGCAGCATTCGCTCCCCCT		(*7*)
*ompB*			
4			
120-M59	CCGCAGGGTTGGTAACTGC	862	(*8*)
120–807	CCTTTTAGATTACCGCCTAA		(*8*)
RR0155-*rpmB*			
5			
Forward	TTTCTAGCAGCGGTTGTTTTATCC	290	(*9*)
Reverse	TTAGCCCATGTTGACAGGTTTACT		(*9*)
RR1240-*tlc5^b^*			
6			
Forward	CGGGATAACGCCGAGTAATA	357	(*10*)
Reverse	ATGCCGCTCTGAATTTGTTT		(*10*)
*cspA-ksgA*			
7			
Forward	CATCACTGCTTCGCTTATTTT	405	(*9*)
Reverse	ATTTCTTTTCTTCCTCTTCATCAA		(*9*)

Whole-body remnants of the 15 ticks used to inoculate shell vials were also subjected to DNA extraction and processed by PCR for the rickettsial *gltA* gene (Table); only 1 tick (the one that provided the rickettsial isolate) contained rickettsial DNA, indicating a 6.6% (1/15) infection rate. We confirmed the taxonomic identification of this tick as *A. patinoi* by generating mitochondrial 16S rRNA partial sequences from it and from an *A. patinoi* paratype that is deposited in the tick collection of the University of São Paulo (Brazil) (accession no. CNC-1585) (*5*). Both sequences were 100% identical to each other (deposited into GenBank under accession nos. KP036466–KP036467).

A 3 mL-aliquot of the second infected cell passage was inoculated intraperitoneally into an adult male guinea pig, in which high fever (rectal temperature >40.0°C) developed during days 5–8 days post inoculation. A total of 3 guinea pig passages were performed, always followed by high fever. A second passage animal survived; scrotal necrosis developed (Technical Appendix Figure 2), and this animal seroconverted to *R. rickettsii* with 32,768 endpoint IgG titer through the immunofluorescence assay, as described (*2*).

A highly pathogenic strain of *R. ricketsii* was isolated from an *A. patinoi* specimen collected at Villeta, where recent human cases of RMSF have been reported (*1*). More than 70 years ago, the only previous *R. rickettsii* tick isolates in Colombia were obtained from *A. cajennense* s.l. in Tobia, only 20 km from Villeta (*4*). At that time, ticks of the *A. cajennense* complex were considered natural vectors of *R. rickettsii* in Tobia (*4*). Because the *A. cajennense* s.l. complex was recently found to be represented in the eastern cordillera of Colombia (which includes Tobia and Villeta) by the species *A. patinoi* (*5*), the tick isolates obtained >70 years ago also are highly likely to have been obtained from *A. patinoi*. Therefore, *A. patinoi* ticks should be considered the main vector of *R. rickettsii* to humans in this region of Colombia.

Technical Appendix Testing of *Rickettsia rickettsii* isolated from an *Amblyomma patinoi* tick, Villeta, Colombia, August 2013.
