# Supplementary material for: Rickettsia rickettsii in Amblyomma patinoi Ticks, Colombia
Source: Emerg Infect Dis. 2015 Mar;21(3):537–9. doi: 10.3201/eid2013.140721 (PMC4344264; doi:10.3201/eid2013.140721)
Supplement: Technical Appendix — Testing of Rickettsia rickettsii isolated from an Amblyomma patinoi tick, Villeta, Colombia, August 2013. [file 14-0721-Techapp-s1.pdf]

Article DOI: http://dx.doi.org/10.3201/eid2103.140721

Rickettsia rickettsii in *Amblyomma patinoi* Ticks, Colombia

Technical Appendix

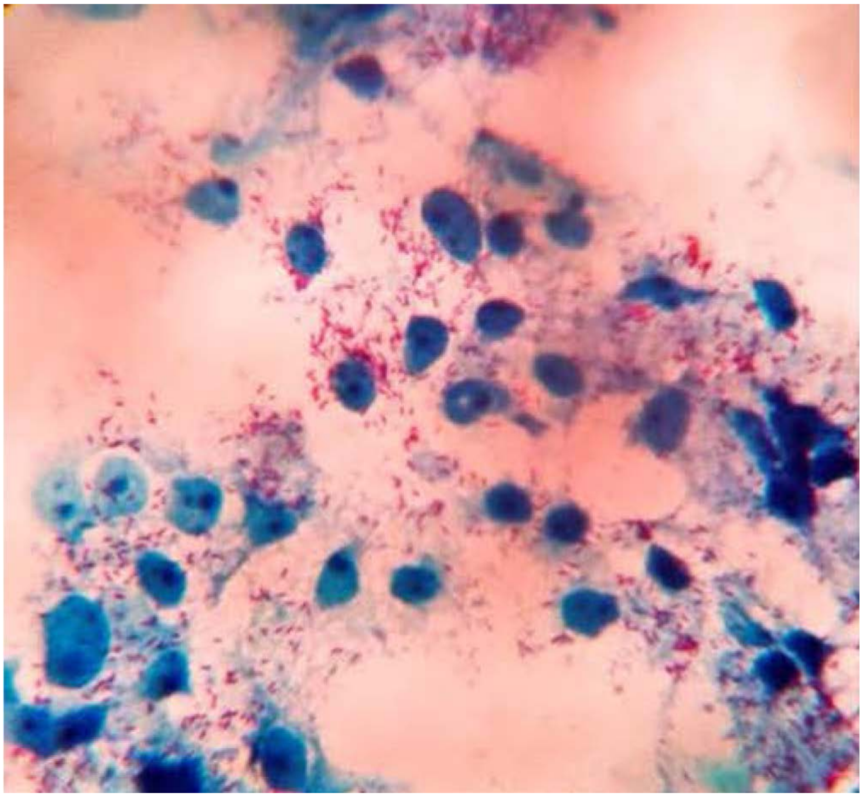

Technical Appendix Figure 1. *Rickettsia rickettsii* isolated from an *Amblyomma patinoi* tick, Villeta, Colombia, August 2013. Second passage of infected Vero cells that were inoculated with infected tick extract through the shell vial technique. Gimenez staining, original magnification ×100.

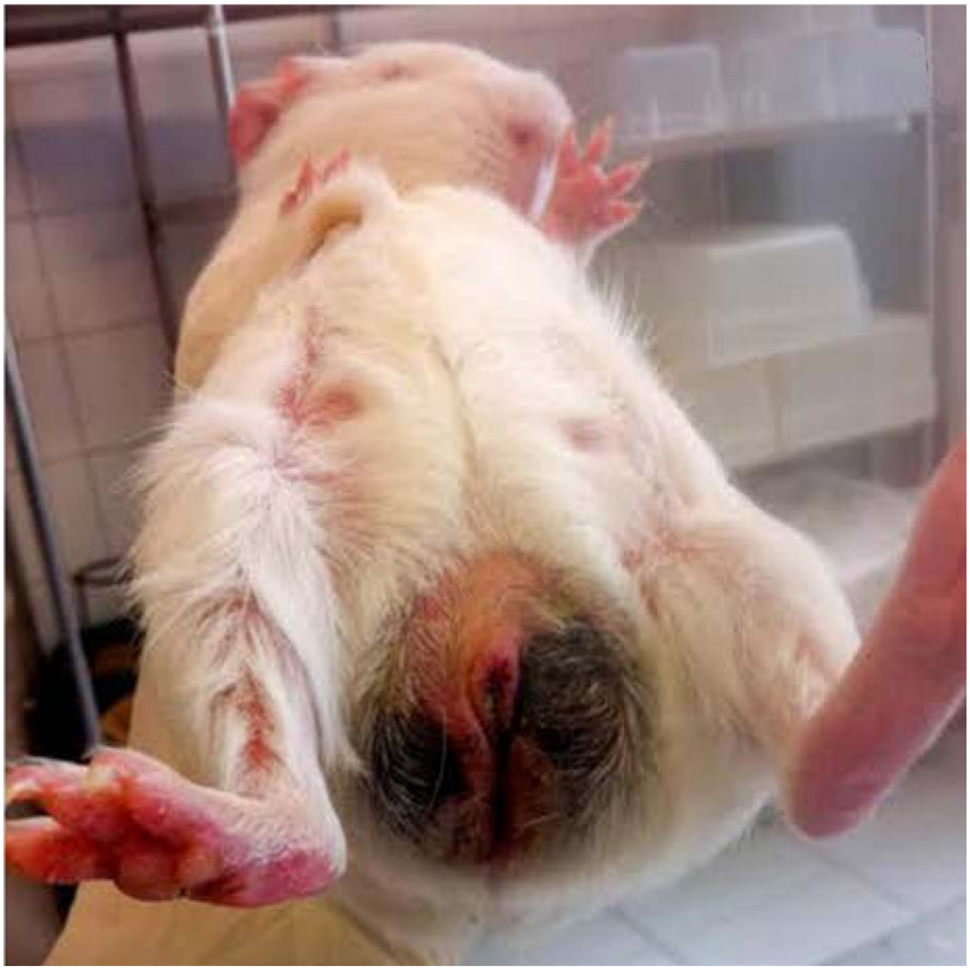

Technical Appendix Figure 2. Scrotal necrosis in a guinea pig that had been inoculated with *Rickettsia rickettsii*–infected Vero cells 14 days previously.
